# Water Splitting Reaction Mechanism on Transition Metal (Fe-Cu) Sulphide and Selenide Clusters—А DFT Study

**DOI:** 10.3390/ma17010056

**Published:** 2023-12-22

**Authors:** Ellie Uzunova, Ivelina Georgieva, Tsvetan Zahariev

**Affiliations:** Institute of General and Inorganic Chemistry, Bulgarian Academy of Sciences, 1113 Sofia, Bulgaria; ivelina@svr.igic.bas.bg (I.G.); tzahariev@svr.igic.bas.bg (T.Z.)

**Keywords:** chalcogenides, ab initio methods, DFT, artificial photosynthesis, transition metal sulfides, selenides, carbonyl complexes

## Abstract

The tetracarbonyl complexes of transition metal chalcogenides M_2_X_2_(CO)_4_, where M = Fe, Co, Ni, Cu and X = S, Se, are examined by density functional theory (DFT). The M_2_X_2_ core is cyclic with either planar or non-planar geometry. As a sulfide, it is present in natural enzymes and has a selective redox capacity. The reduced forms of the selenide and sulfide complexes are relevant to the hydrogen evolution reaction (HER) and they provide different positions of hydride ligand binding: (i) at a chalcogenide site, (ii) at a particular cation site and (iii) in a midway position forming equal bonds to both cation sites. The full pathway of water decomposition to molecular hydrogen and oxygen is traced by transition state theory. The iron and cobalt complexes, cobalt selenide, in particular, provide lower energy barriers in HER as compared to the nickel and copper complexes. In the oxygen evolution reaction (OER), cobalt and iron selenide tetracarbonyls provide a low energy barrier via OOH* intermediate. All of the intermediate species possess favorable excitation transitions in the visible light spectrum, as evidenced by TD-DFT calculations and they allow photoactivation. In conclusion, cobalt and iron selenide tetracarbonyl complexes emerge as promising photocatalysts in water splitting.

## 1. Introduction

Transition metal chalcogenides as clusters, layers or coordination compounds with various ligands possess the unique property to accept, store and donate electrons to substrates. Coordination of cyanide, carbonyl or more complex protein-like ligands, strongly influences the electron distribution within these clusters and enables their application in redox catalysis, including water splitting and carbon dioxide reduction. Water dissociation to hydrogen and oxygen, 2H_2_O(g) → 2H_2_(g) + O_2_(g), is a strongly endothermic process with an enthalpy of 483.7 kJ mol^−1^ at 298 K [1]. In addition to the high endothermic effect, reaction barriers certainly add to the overall energy needed to split water. The electrochemical route requires a four-electron transfer for the cathode reaction delivering hydrogen 4H^+^ + 4e^−^ → 2H_2_(g), and the anode reaction delivering oxygen, 2H_2_O → 2O_2_(g) + 4H^+^ + 4e^−^. Numerous studies were focused on the optimization of the electrochemical reaction, its pH dependence and the well-known overpotential in the oxygen evolution reaction (OER) [2,3,4,5,6,7]. The OER and the O–O bond formation chemistry was explored using a variety of materials: theoretical studies on cobalt oxide clusters and on photosystem II, which includes a manganese complex [3,4], and heterogeneous metallic electrocatalysts [5,6]. The importance of an oxygenated intermediate after [H^+^,e^−^] removal was outlined [3,4,5,6,7,8]. The research efforts have targeted the analogues of natural enzymes, used in photosynthesis, in order to obtain efficient photocatalysts and overcome the high reaction barriers [9,10,11,12,13]. The photocatalysts can either be applied directly as electrode materials, or used as bulk materials in a photoelectrochemical cell. Theoretical studies help in preliminary studies for discerning promising photocatalytic materials and in the elucidation of the reaction mechanism.

As structural analogs of ferredoxin and hydrogenase enzymes, the sulfides of iron and nickel, with carbonyl or halogen ligands, have been the subject of experimental and theoretical studies [10,11,12,13,14,15]. Despite the promising results on hydrogen evolution by photoactivated Fe_2_S_2_(CO)_6_ complexes [10], other transition metal chalcogenide complexes received much less attention. The present study simulates enzyme analogs of the transition metals, Fe, Co, Ni and Cu, which form binuclear clusters M_2_X_2_ (M = Fe, Co, Ni, Cu and X = S, Se) and they are coordinated by carbonyl ligands so as to form symmetric tetracarbonyl complexes. The electronic structure of these complexes is examined by density functional theory and the reaction of water splitting is traced by transition state theory. The catalytic pathway with the possibility of photoactivation of the two half-reactions: Hydrogen Evolution Reaction (HER) and Oxygen Evolution Reaction (OER) are traced by time-dependent density functional theory (TD-DFT).

## 2. Materials and Methods

All calculations were performed with the B3LYP functional [16,17,18,19], which includes local and non-local terms as implemented in the Gaussian 16 package [20]. The standard 6-311+G(2df) basis set with diffuse and polarization functions was employed, which consists of the McLean–Chandler (12s, 9p) → (621111,52111) basis sets for second-row atoms and the Wachters–Hay all-electron basis set for the first transition row, using the scaling factors of Raghavachari and Trucks [21,22,23,24,25]. In their ground states, selenium-containing clusters were reoptimized using the QZVP basis set [26,27], but no significant change in bond lengths (within 1.6%) or relative energies (within 0.9%) occurred. The differences obtained with the LanL2DZ basis [28,29,30] were even smaller, differing by only 0.7% regarding bond lengths and by 0.9% regarding relative energies. The selection of the density functional and basis set was based on calculations of the diatomic molecules and the diiron disulfide hexacarbonyl complex from previous studies [31,32,33], where different density functionals were compared, as there are sufficiently accurate experimental data for these compounds. Proton and electron affinities are calculated as the energy required to attach a proton or electron, respectively. For proton–electron couples a subsequent proton and electron attachment are calculated.

The spin-unrestricted formalism was applied and calculations in the broken symmetry (BS) approach were performed, which consists of the localization of the opposite spins on different parts of the molecule to give a mono-determinant representation of the spin exchange interactions, thus reducing the symmetry of the space and spin wavefunctions with respect to that of the nuclear framework [31,32,33,34]. The synchronous transit-guided quasi-Newton (STQN) method was used for the transition state optimizations [35,36]. Intrinsic reaction coordinate (IRC) calculations were performed to confirm the transition state structures and to evaluate activation energies [37,38]. Reaction studies using water as a solvent were performed using the Polarizable Continuum Model [39] (PCM). Time-dependent (TD) DFT was used [40,41] to determine the excitation energies of the ground state cluster complexes, the reaction intermediates and oscillator strengths. Dispersion effects were taken into account for the ground states and the reaction intermediates by using the formula of Grimme with Becke–Johnson damping [42]. The bond populations and charge distributions were examined by using natural orbitals and natural bond orbital (NBO) analysis [43,44].

## 3. Results and Discussion

### 3.1. Structure and Bonding of the Tetracarbonyl Complexes of Metal Disulfides and Diselenides, M_2_X_2_(CO)_4_

**Figure 1:**
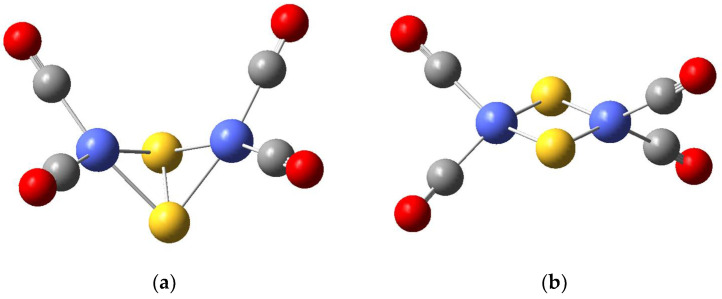
(**a**) The global minimum of Co_2_S_2_(CO)_4_ in rhombic non-planar configuration. (**b**) Co_2_S_2_(CO)_4_ with a planar core. Legend: Co atoms are blue, sulfur atoms—yellow; carbon atoms—grey; oxygen atoms—red.

The tetracarbonyl complexes of cobalt, iron and nickel disulfides possess two conformations of the core M_2_X_2_: rhombic and planar; see Figure 1. The global energy minimum structures of all M_2_S_2_(CO)_4_ complexes (M = Fe, Co, Ni, Cu) have a rhombic non-planar core M_2_X_2_. The energy gap between non-planar and planar configurations is 67 kJ mol^−1^ for Fe_2_S_2_(CO)_4_ and 88 kJ mol^−1^ for Co_2_S_2_(CO)_4_. It is much smaller for Ni_2_S_2_(CO)_4_, 21 kJ mol^−1^. The selenide complexes M_2_Se_2_(CO)_4_ (M = Fe, Co, Cu) form only a non-planar rhombic core M_2_Se_2_, whereas Ni_2_Se_2_(CO)_4_ is found as a planar and non-planar structure, the planar being the global minimum and the rhombic one lying by 39 kJ mol^−1^ higher in energy. All of the sulfides and selenides with rhombic non-planar structures contain an S-S or Se-Se bond.

According to our results, the geometry flexibility of the metal disulfide and diselenide core matters to the reactivity of these complexes by providing a favorable orientation towards substrate molecules. Thus, water adsorption proves to be an exothermic process; however, the adsorption energy depends on the core configuration: on the planar clusters, the heat of adsorption is 25–30 kJ mol^−1^, while on the non-planar clusters, it is weaker, at 8–13 kJ mol^−1^. Further, in the subsequent reaction steps (dissociation, HER, OER), the active site M_2_X_2_ may present variable deviation from planarity.

**Figure 2:**
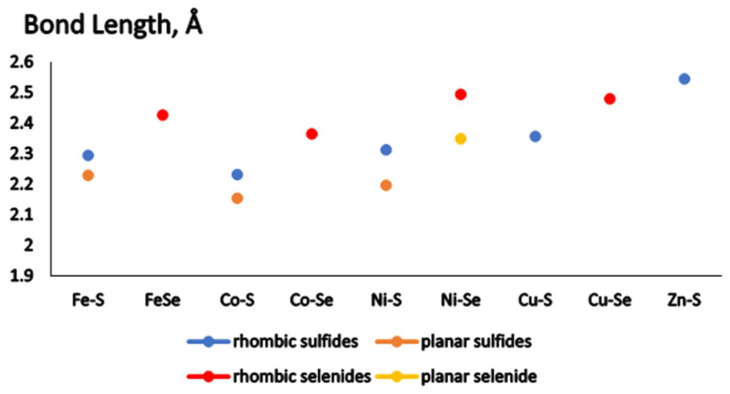
Metal cation-to-chalcogenide bond lengths for M_2_X_2_(CO)_4_ complexes, M = Fe, Co, Ni, Cu, Zn; X = S, Se.

For all M_2_X_2_(CO)_4_ complexes, which have two conformations (planar and non-planar), the M-X bond lengths in the planar core are shorter than in the non-planar rhombic core, as shown in Figure 2. The Fe-S and Co-S bond lengths variation is much smaller as compared to the Ni-S or Ni-Se bond lengths difference. For M_2_X_2_(CO)_4_ complexes studied, the Co-S/Se bond length is the shortest one and this result corresponds to the strong Co-S bond found in the diatomic molecule [45]. For the global minimum structures of M_2_X_2_(CO)_4_, the bond lengths increase as follows: Co-S < Fe-S < Ni-S < Cu-S < Co-Se < Fe-Se < Cu-Se < Ni-Se < Zn-S. While zinc sulfide exists in the solid state and forms clusters without ligands, the filled 3d shell does not allow interaction with a strong electron donor as the carbonyl groups. The Zn-CO bonds reach 2.183 Å, while in the other complexes, they do not exceed 1.95 Å. The Zn-S bonds are also lengthened to 2.541 Å. Thus, Zn_2_S_2_(CO)_4_ is not examined further in the present study.

**Figure 3:**
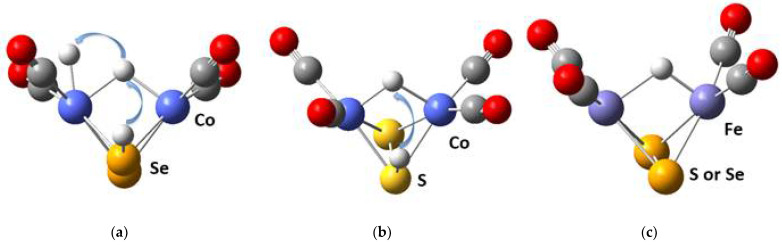
(**a**) The three distinct positions of hydride in Co_2_Se_2_(CO)_4_. (**b**) The two distinct positions of hydride in Co_2_S_2_(CO)_4_. (**c**) The single position of hydride in Fe_2_S_2_(CO)_4_ valid also for Fe_2_Se_2_(CO)_4_. Legend as in Figure 1.

The calculated proton affinities of the sulfide complexes M_2_S_2_(CO)_4_ complexes are higher than the proton affinities of the corresponding selenide complexes Table 1. The chalcogenide complexes of Cu and Ni have markedly higher proton affinities as compared to the chalcogenides of Fe and Co, but subsequent electron addition with the formation of proton–electron couples [H^+^,e^−^] indicates lower affinities for Ni_2_Se_2_(CO)_4_, Cu_2_S_2_(CO)_4_ and particularly for Cu_2_Se_2_(CO)_4_. The protons are always attached at the chalcogenide center (S, Se), while the hydride ligand formed upon neutralization of the positive charge (H^+^) can either remain located at the chalcogenide center or bind at the metal cation sites. Usually, the hydride ligand is centered between the metal cations and forms equal M-H-M bonds, and this was experimentally proven in diiron disulfide complexes, but configurations with a single M-H bond by binding predominantly at one cation site are also possible [9,31,32,33]. The values of the proton–electron affinities for different sites allow us to discern the stability of configurations with hydride ligands, as shown in Figure 3. In iron sulfides and selenides, protonation occurs at a chalcogenide site, and subsequent reduction shifts the hydride ligand to a single stable position with equal Fe-H bond lengths, as shown in Figure 3c. In nickel and copper sulfides and selenides, the hydride ligand remains bonded at the chalcogenide site. Only cobalt centers provide several stable sites for the coordination of a hydride ligand: S-H (Se-H) and Co-H-Co for the disulfides and diselenides, as well as a stable site Co-H, available only in the diselenide complex, as shown in Figure 3a. The role of hydride ligands is crucial for the hydrogen evolution reaction and for the redox capacity in other reactions such as carbon dioxide reduction.

The rhombic core efficiently redistributes the positive charges induced by the binding of protons, substrates, or electron density from electron-donor ligands. The bare chalcogenide rhombic clusters M_2_X_2_ with M = Fe, Co, Ni, Cu and X = S, Se, are structural analogs of the corresponding oxide clusters, with planar configuration in their global minima, stabilized by antiferromagnetic coupling [46,47,48,49]. They readily coordinate electron-donor ligands, e.g., water molecules, halogen ligands and carbonyl groups [15,46,47]. Up to six carbonyl groups can be attached, with three at each cation site [10,11,12,13,14,50]. The loss of a carbonyl ligand from Fe_2_S_2_(CO)_6_ requires 154 kJ mol^−1^ and, similarly, for Co_2_S_2_(CO)_6_ the energy needed is 151 kJ mol^−1^, but the selenide complexes have low stability as hexacarbonyls: our calculations indicate that Co_2_Se_2_(CO)_6_ loses a carbonyl ligand by only 12 kJ mol^−1^. The tetracarbonyl complexes proved stable for both the sulfide and the selenide complexes—the loss of a carbonyl ligand from Co_2_Se_2_(CO)_4_ requires 197 kJ mol^−1^. It is thus useful to compare the electronic structure properties of the hexacarbonyl and tetracarbonyl complexes of cobalt, which form the shortest Co-S and Co-Se bonds. In their global minima, the hexacarbonyl and tetracarbonyl complexes contain a non-planar Co_2_X_2_ core with S-S or Se-Se bond, denoted as Co_2_(X_2_), as shown in Figure 1a, Table 2. The metal cation-to-carbon bonds are of typical lengths within 1.75–1.82 Å. Natural orbital analysis reveals that in disulfides, cobalt valence orbital occupancies vary in the frame Co 4s(0.43–0.46) 3d(8.32–8.49) 4p(0.80–0.90) and this applies for both the tetracarbonyl and hexacarbonyl complexes, Co_2_(S_2_)(CO)_6_ and Co_2_(S_2_)(CO)_4_. The electron density on cobalt centers is thus significantly increased, as compared to the 3d^7^ electron configuration of Co(II). The 4p orbital population indicates 4s3d4p hybridization on cobalt. The sulfide and selenide centers also act as ligands, but the population of the sulfur valence levels is slightly higher than it is on selenium, namely S 3s(1.75) 3p(3.90–4.30) and Se 4s(1.75)4p(3.70–3.91). Cobalt centers in diselenide complexes increase their local valence orbital population, respectively, to Co 4s(0.45–0.47) 3d(8.4–8.6)4p(1.15–1.23). In the presence of a hydride ligand, the H 1s orbital population is between 0.83–1.01 and corresponds to a hydrogen atom, 1s(1), but with a partial positive charge, or minor negative for 1s(1.01) in [Co_2_HS_2_](CO)_6_, Table 2. Among the cobalt disulfide complexes, the HOMO(SOMO)-LUMO gaps are higher for the hexacarbonyls than they are for the tetracarbonyls. The attachment of a hydride ligand increases the HOMO(SOMO)-LUMO gap in the hexacarbonyls, but in tetracarbonyls, the binding of a hydride ligand at a chalcogenide center (S, Se) always lowers the HOMO(SOMO)-LUMO energy gap. When the hydride ligand binds to the cobalt centers, the SOMO-LUMO gap in the tetracarbonyls strongly increases. In Co_2_(Se_2_)(CO)_4_, the HOMO-LUMO energy gap is higher than in the disulfide complexes. The binding of hydride in a midway position between cobalt centers, Figure 2, increases the SOMO-LUMO gap in [Co_2_HS_2_](CO)_6_ by 0.26 eV relative to the Co_2_(S_2_)(CO)_6_, while in the corresponding tetracarbonyl, [(Co_2_H)S_2_](CO)_4_, it decreases by 0.21 eV relative to the Co_2_(S_2_)(CO)_4_ complex. The formation of a single Co-H bond, which is observed only in selenide complexes, e.g., [Co(Co-H)Se_2_](CO)_4_, increases the SOMO-LUMO gap by only 0.04 eV relative to Co_2_(Se_2_)(CO)_4_, but this is the highest energy gap observed among the sulfide and selenide complexes with or without hydride ligand.

According to the TD-DFT calculations, the protonated and reduced cobalt complexes possess intense light-absorption bands in the visible part of the spectrum, as shown in Table 3. Typically, the bands are blue shifted for the selenide complexes when analogous conformations are compared. The TD-DFT calculated highest intensity bands in the UV-Vis spectra correspond to multiple vertical electron excitations within the Co_2_X_2_ core, with dominant Co → X and Co → H transitions, or metal to ligand charge transfer bands, MLCT. Though the hydride-bonded complexes are powerful reducing agents even without photoactivation, the presence of intense bands in the visible region allows excitation and further enhancement of reactivity towards inert molecules such as CO_2_.

### 3.2. The Hydrogen Evolution Reaction (HER) on Tetracarbonyl Complexes of Metal Disulfides and Diselenides, M_2_X_2_(CO)_4_

**Figure 4:**
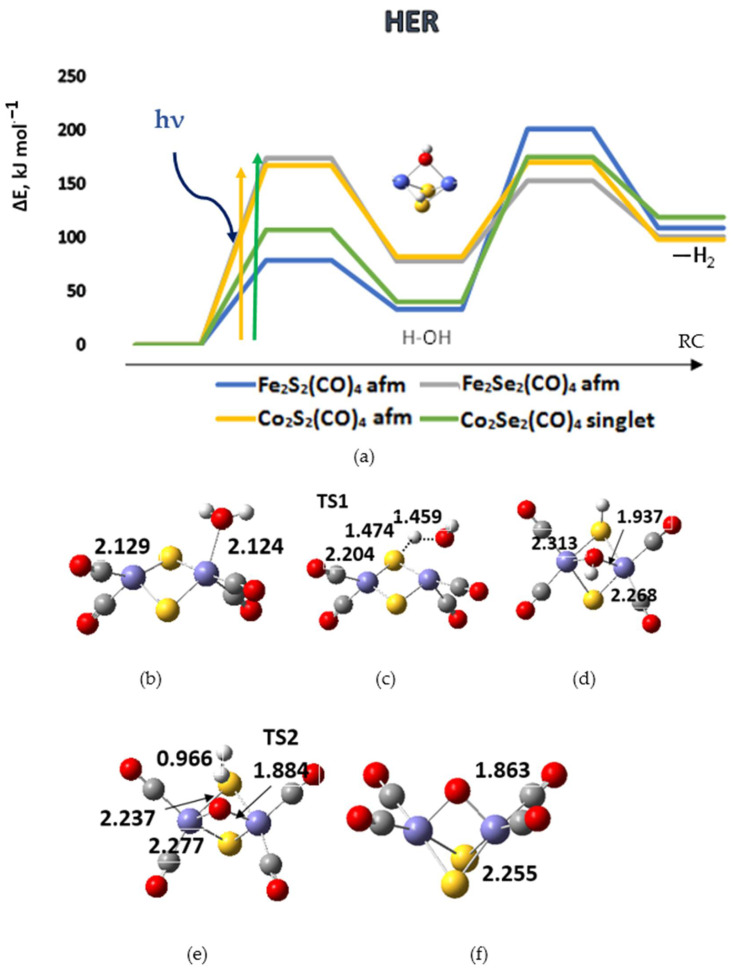
(**a**) The reaction path of water dissociation and hydrogen evolution (HER) on different tetracarbonyl complexes of iron and cobalt. TS1 corresponds to the reaction barrier of primary dissociation, and TS2 to dihydrogen formation. afm denotes antiferromagnetic singlet ground states. ΔE is the energy difference relative to the ground state complexes; RC—reaction coordinate. The excitation energies of representative most intense singlet-singlet transitions induced by light absorption are denoted by vertical arrows. (**b**) Structure of the water adsorption complex on Fe_2_S_2_(CO)_4_; (**c**) structure of TS1; (**d**) dissociated water on Fe_2_S_2_(CO)_4_; (**e**) structure of TS2; (**f**) structure of the product with bridging oxygen after hydrogen desorption. Cartesian coordinates of intermediate species are presented in Supporting Information.

The reaction path in water dissociation with hydrogen evolution includes an intermediate step of breaking an H-OH bond with the formation of an S-H or Se-H bond and a bridging hydroxyl group, as shown in Figure 4.

In a photoactivated reaction, the following elementary steps are followed:

[M_2_X_2_](CO)_4_ + H_2_O + hν ⇒ [M_2_X_2_H*](CO)_4_ + OH* ⇒ [M_2_(OH)X_2_H](CO)_4_ ⇒ [M_2_OX_2_](CO)_4_ + H_2_ (1)

**Figure 5:**
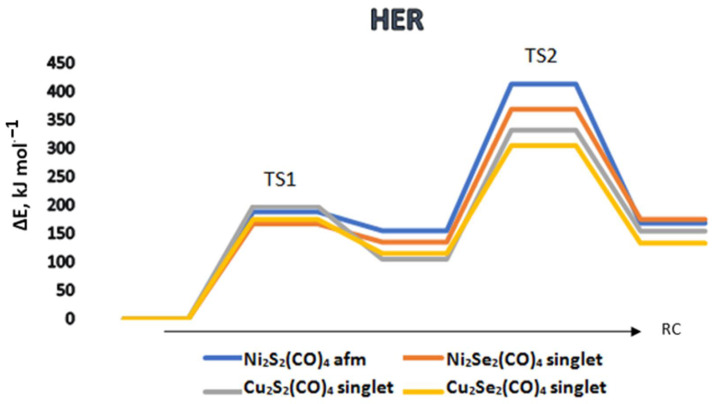
The reaction path of water dissociation and hydrogen evolution (HER) on different tetracarbonyl complexes of nickel and copper. TS1 corresponds to the reaction barrier of primary dissociation, and TS2 to dihydrogen formation. afm denotes antiferromagnetic singlet ground states. ΔE is the energy difference relative to the ground state complexes; RC—reaction coordinate.

The reaction mechanism was traced for the global minima of the complexes, which correspond to either a diamagnetic (*d*) singlet or antiferromagnetic (*afm*) singlet ground states. The chalcogenide complexes of cobalt and iron are presented in Figure 4. Dicobalt diselenide, Co_2_Se_2_(CO)_4_ (*d*) and diiron disulfide, Fe_2_S_2_(CO)_4_ (*afm*) provide a more favorable energy path in the first reaction step of water dissociation, but in the following step of dihydrogen formation, the corresponding energy barriers are with the highest values. The reverse case is observed with Fe_2_Se_2_(CO)_4_ (*afm*), and though it reaches the highest energy barrier in the first reaction step, in the next step of dihydrogen formation, it provides the lowest energy path. Overall, it may be concluded that Co_2_Se_2_(CO)_4_, (*d*), Co_2_S_2_(CO)_4_ (*afm*), and Fe_2_Se_2_(CO)_4_ (*afm*) perform best: for Co_2_Se_2_(CO)_4_, the first reaction barrier is 107 kJ mol^−1^ and the second reaction barrier is 135 kJ mol^−1^, whereas the lowest value for the second step of hydrogen formation is for Fe_2_Se_2_(CO)_4_, 75 kJ mol^−1^, preceded by a barrier of 174 kJ mol^−1^. Co_2_S_2_(CO)_4_ stays between these values—the first barrier at 167 kJ mol^−1^ and the second barrier at 88 kJ mol^−1^. The chalcogenide complexes of the remaining elements—nickel and copper perform worse in the water dissociation and HER, as shown in Figure 5. The lowest energy barrier for the first step is 167 kJ mol^−1^ for Ni_2_Se_2_(CO)_4_, followed by 369 kJ mol^−1^ for dihydrogen formation. Among these complexes, Cu_2_Se_2_(CO)_4_ performs best, with a first reaction step barrier of 175 kJ mol^−1^ and a second barrier of 305 kJ mol^−1^. Though the reaction barriers for the second reaction step look prohibitively high, the role of the first step of water dissociation is important, as pointed out in studies on carbon dioxide trapping and activation [51,52]. It provides surface hydroxyl or sulfonyl groups, which are able to activate the CO_2_ molecule and promote in this way carboxyl or carbonate formation.

In addition, the triplet potential energy surfaces of the complexes M_2_X_2_(CO)_4_ were also examined as all of them have stable triplet states (local minima); see Supporting Information (SI). All of the triplet states are higher in energy than the singlet ground states (by 47 ÷ 152 kJ mol^−1^) and the energy gaps between the triplet and singlet state minima are presented in Table S1 in SI. The calculated triplet HER pathway of chalcogenide complexes of cobalt and iron in Figure S1 (SI) showed that the lowest energy barrier in the first step is provided by Co_2_Se_2_(CO)_4_, but in the following step of dihydrogen formation, it reaches the highest energy value of 213 kJ mol^−1^. This triplet state was found to be 68 kJ mol^−1^ higher in energy than the singlet ground state. The triplet state reaction path does not provide a lowering of the second reaction barrier, which is rate determining. The triplet state reaction path for nickel and iron chalcogenide complexes is highly unfavorable: the lowest energy barrier for the rate-determining step is 670 kJ mo^−1^ for Ni_2_Se_2_(CO)_4_, see Figure S2 in SI.

The activation barriers in HER differ largely depending on the type of system studied [53,54,55,56] and the calculated values for TS1 varied in the range of 40–210 kJ mol^−1^ for (MO_2_)_n_ clusters (M = Ti, Zr, Hf, n = 1–3) [56]. The barriers for TS2 were not much larger, ranging from 63 to 210 kJ mol^−1^. A systematic dependence on the type and size of the clusters was not reported. Our results on the chalcogenides of cobalt and iron fall within a similar range, 68–174 kJ mol^−1^ for TS1 and 88–213 kJ mol^−1^ for TS2 and we also observe a lack of systematic change depending on the composition of the complexes.

### 3.3. The Oxygen Evolution Reaction (OER) on Tetracarbonyl Complexes of Metal Disulfides and Diselenides, M_2_X_2_(CO)_4_

**Figure 6:**
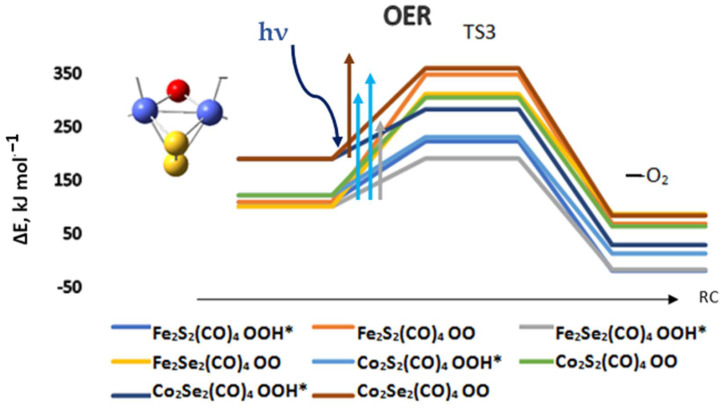
The reaction path of oxygen formation and oxygen evolution for cobalt and iron chalcogenide complexes. ΔE is the energy difference relative to the ground state complexes; RC—reaction coordinate. The global minima of the complexes were used, as denoted in Figure 4. The excitation energies of representative most intense transitions induced by light absorption are denoted by vertical arrows.

Previous studies outlined the role of pH and oxygenated reaction intermediates after proton–electron removal [4,5,6,7,8,53,54,55]. The oxygen evolution reaction on the chalcogenide complexes also proved to be pH dependent and in acidic media it proceeds via protonation and a peroxo intermediate OOH*, which is the more favorable route, Equation (2). Here again, the lowest energy barriers of 91 and 93 kJ mol^−1^ are obtained for the selenide complexes, Fe_2_Se_2_(CO)_4_, and Co_2_Se_2_(CO)_4_, followed by the disulfides of iron and cobalt, Fe_2_S_2_(CO)_4_ and Co_2_S_2_(CO)_4_ with barriers of 114 and 109 kJ mol^−1^, as shown in Figure 6. The OER reaction begins with an oxygen-bridged complex, which comes out from the HER reaction.

[M_2_OX_2_](CO)_4_ + O[H^+^,e^−^] + hν ⇒ [M_2_(OOH)X_2_](CO)_4_ ⇒ [M_2_HX_2_](CO)_4_ + O_2_ (2)

The reaction barriers for OER are thus not prohibitively high, but the presence of favorable light absorption bands would certainly allow a photocatalytic pathway. TD-DFT calculations indeed indicate the presence of intense bands in the visible spectrum for the dioxygen intermediates and for the peroxo intermediates, Table 4. They are slightly blue-shifted, as compared to the reduced forms of the corresponding complexes. The energy provided by light absorption is sufficient to provide activation relevant to the reaction barriers of OER via OOH*, and for the high-lying barriers of dioxygen formation. Among the most favorable energy pathways for OER, as reported in the literature, is that on a molecular cubane complex, and a reaction barrier of 84 kJ mol^−1^ was experimentally determined [8]. Theoretical modeling with small cobalt oxide clusters provided an accurate estimate of this barrier and the reported calculated value is 97 kJ mol^−1^ [4]. The cobalt and iron chalcogenides thus provide comparable reaction barriers, according to our results, via the peroxo intermediate OOH*, as shown in Figure 6. The OER can be started from the triplet state of the complexes, but the resulting activation barriers are higher by 87–150 kJ mol^−1^; see Figure S3 in SI.

In a pH-neutral or alkaline solvent, Co_2_Se_2_(CO)_4_ and Co_2_S_2_(CO)_4_ provide the lowest energy path for dioxygen formation with barriers of 170 and 183 kJ mol^−1^, respectively. The reaction pathway in this case is the following Equation (3):

[M_2_OX_2_](CO)_4_ + 2OH* + hν ⇒ [M_2_(OO)X_2_](CO)_4_ + [H^+^,e^−^]-OH ⇒ [M_2_X_2_](CO)_4_ + H_2_O + O_2_ (3)

Both reaction paths include proton–electron transfer. The light absorption bands correspond to electron excitations within the M_2_X_2_ core, M → X of MLCT character, but they include the bonded dioxygen species with transitions O → M, which correspond to ligand-to-metal charge transfer (LMCT). This is another proof of the great capacity of the M_2_X_2_(CO)_4_ complexes to redistribute electron density. The transitions are of the type triplet to triplet for Equation (3) and doublet to doublet for Equation (2).

**Figure 7:**
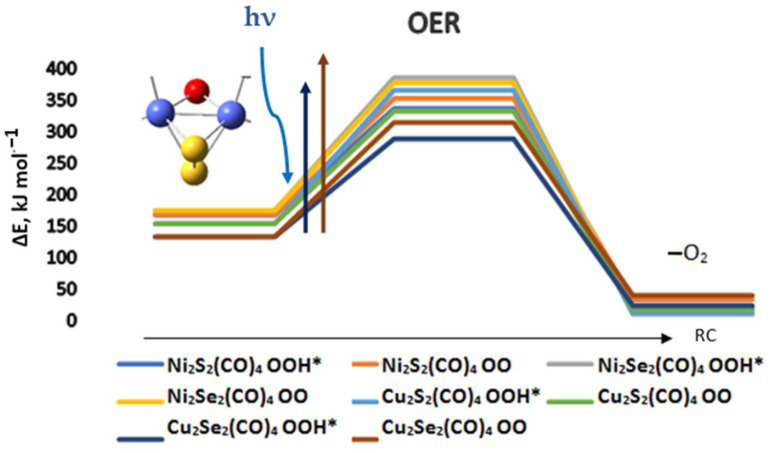
The reaction path of oxygen formation and oxygen evolution for nickel and copper chalcogenide complexes. ΔE is the energy difference relative to the ground state complexes; RC—reaction coordinate. The global minima complexes were used, as denoted in Figure 5. The excitation energies of representative most intense transitions induced by light absorption are denoted by vertical arrows.

For the complexes of nickel and copper, the role of acidity is not pronounced, as shown in Figure 7. The lowest reaction barrier is indeed for OOH* formation on Cu_2_Se_2_(CO)_4_ and it is 156 kJ mol^−1^, followed by OOH* formation on Ni_2_S_2_(CO)_4_ with a barrier of 169 kJ mol^−1^. The formation of dioxygen on Ni_2_S_2_(CO)_4_ does not change the barrier significantly—it goes up to 185 kJ mol^−1^. On Ni_2_Se_2_(CO)_4_ the barrier heights for OOH* and OO formation are reversed: dioxygen formation requires 203 kJ mol^−1^, while the pathway via OOH* intermediate goes through a slightly higher barrier of 211 kJ mol^−1^. The OER can be started from the triplet state of the complexes, but the resulting activation barriers are higher by 157–250 kJ mol^−1^ higher; see Figure S3 in SI.

## 4. Conclusions

The chalcogenide tetracarbonyl complexes of the 3d transition metal elements (Fe-Cu) follow a pathway with similar intermediates in the reaction of water splitting, with low energy barriers for the singlet pathway, and the presence of visible light-absorption bands favor photoactivation. Though only sulfides are direct structural analogs of natural enzymes, selenides have similar proton affinities, proton–electron affinities, and light absorption bands and may outperform sulfides in the OER reaction. Cobalt and iron sulfides and selenides perform better than the corresponding complexes of nickel and copper for both the HER and OER reactions. Protonation affects positively the energy barriers for OER in the case of cobalt and iron chalcogenide complexes, but the effect is weaker for the nickel and copper analogs. The hydride intermediates, relevant to hydrogen evolution, and also the oxidized intermediates possess favorable light absorption bands in the visible spectrum. They allow photoactivation in the complexes, for which the reaction barriers are high.

## Figures and Tables

**Table 1:** Calculated proton affinities (PA, kJ mol^−1^) and [H^+^,e^−^] affinities (kJ mol^−1^) for [M2-X2] tetracarbonyls, M = Fe, Co, Ni, Cu and X = S, Se.

Cluster/Binding	PA	[H^+^,e^−^]
[Fe_2_S_2_H]^+^(CO)_4_; S-H	651	
[Fe_2_HS_2_](CO)_4_; Fe-H-Fe		229
[Fe_2_Se_2_H]^+^(CO)_4_; Se-H	639	
[Fe_2_HSe_2_](CO)_4_; Fe-H-Fe		228
[Co_2_S_2_H]^+^(CO)_4_; [Co_2_S_2_H](CO)_4_; S-H	767	224
[Co_2_HS_2_](CO)_4_; Co-H-Co		177
[Co_2_Se_2_H]^+^(CO)_4_; [Co_2_Se_2_H](CO)_4_; Se-H	748	191
[Co_2_HSe_2_](CO)_4_; Co-H-Co		179
[Co_2_HSe_2_](CO)_4_; Co-H		201
[Ni_2_S_2_H]^+^(CO)_4_; [Ni_2_S_2_H](CO)_4_; S-H	935	181
[Ni_2_Se_2_H]^+^(CO)_4_; [Ni_2_Se_2_H](CO)_4_; Se-H	907	168
[Cu_2_S_2_H]^+^(CO)_4_; [Cu_2_S_2_H](CO)_4_; S-H	968	134
[Cu_2_Se_2_H]^+^(CO)_4_; [Cu_2_Se_2_H](CO)_4_; Se-H	936	81

**Table 2:** HOMO(SOMO)-LUMO (H-L) energy gaps (eV) and electron distribution on the hydride ligand, in cobalt tetracarbonyl and hexacarbonyl complexes calculated by natural population analysis.

Hexacarbonyl Complexes	H-L	H 1s	Tetracarbonyl Complexes	H-L	H 1s	Tetracarbonyl Complexes	H-L	H 1s
Co_2_(S_2_)(CO)_6_	2.84		Co_2_(S_2_)(CO)_4_	2.83		Co_2_(Se_2_)(CO)_4_	3.29	
[Co_2_S_2_H](CO)_6_	2.91	0.89	[Co_2_S_2_H](CO)_4_	1.67	0.87	[Co_2_Se_2_H](CO)_4_	1.56	0.91
[Co_2_HS_2_](CO)_6_	3.10	1.01	[(Co_2_H)S_2_](CO)_4_	2.62	0.86	[(Co_2_H)Se_2_](CO)_4_	2.31	0.85
						[Co(Co-H)Se_2_](CO)_4_	3.33	0.83

**Table 3:** TD-DFT results for protonated and reduced (H^+^,e^−^) cobalt sulfides and selenides. The most intense light absorption bands listed.

Complex	Light Absorption Bands, nm	Oscillator Strength
[Co_2_(S_2_H)]^+^(CO)_4_	703; 910	0.0012; 0.0130
[Co_2_S-SH](CO)_4_	689; 824	0.0060; 0.0010
[Co-H-CoS_2_](CO)_4_	750; 803	0.0020; 0.0040
[Co_2_(Se_2_H)]^+^(CO)_4_	519; 753	0.0028; 0.0036
[Co_2_Se-SeH](CO)_4_	628; 749	0.0011; 0.0017
[Co-H-CoSe_2_](CO)_4_	748; 1610	0.0006; 0.0250
[Co(Co-H)Se_2_](CO)_4_	546; 639	0.0017; 0.0024

**Table 4:** TD-DFT results for OER intermediates of selected sulfide and selenide tetracarbonyl complexes. The most intense light absorption bands are listed.

Complex	Light Absorption Bands, nm	Oscillator Strength
Co_2_S_2_(CO)_4_; O-O	651	0.0014
Co_2_Se_2_(CO)_4_; O-O	582	0.0091
Fe_2_Se_2_(CO)_4_; O-O	807	0.0131
Cu_2_Se_2_(CO)_4_; O-O	430	0.0094
Co_2_S_2_(CO)_4_; OOH*	507; 573	0.0023; 0.0027
Co_2_Se_2_(CO)_4_; OOH*	531	0.0014
Fe_2_Se_2_(CO)_4_; OOH*	798	0.0052
Cu_2_Se_2_(CO)_4_; OOH*	590	0.0130
Ni_2_Se_2_(CO)_4_; OOH*	558	0.0023

## Data Availability

Data are contained within the article and Supplementary Materials.

## Supplementary Materials

The following supporting information can be downloaded at: https://www.mdpi.com/article/10.3390/ma17010056/s1, Cartesian coordinates of the complexes presented in Figures S1, S3, S4b,c,d,e,f. HER/OER reaction path on the triplet potential energy surface, as Figures S1–S3 and singlet to triplet excitation energies as Table S1.
