# Supplementary material for: Water Splitting Reaction Mechanism on Transition Metal (Fe-Cu) Sulphide and Selenide Clusters—А DFT Study
Source: Materials (Basel). 2023 Dec 22;17(1):56. doi: 10.3390/ma17010056 (PMC10780287; doi:10.3390/ma17010056)
Supplement: Supplementary file 1 [file materials-17-00056-s001.zip › materials-2748679-supplementary.pdf]

Supporting Information

Water splitting reaction mechanism on transition metal (Fe-Cu) sulphide and selenide Clusters - a DFT study

Ellie Uzunova [1,*], Ivelina Gergieva [1] and Tsvetan Zahariev [1]

[1] *Institute of General and InorganiC Chemistry, Bulgarian Academy of Sciences, lll3 Sofia, Bulgaria*
* Correspondence: ellie@svr.igic.bas.bg

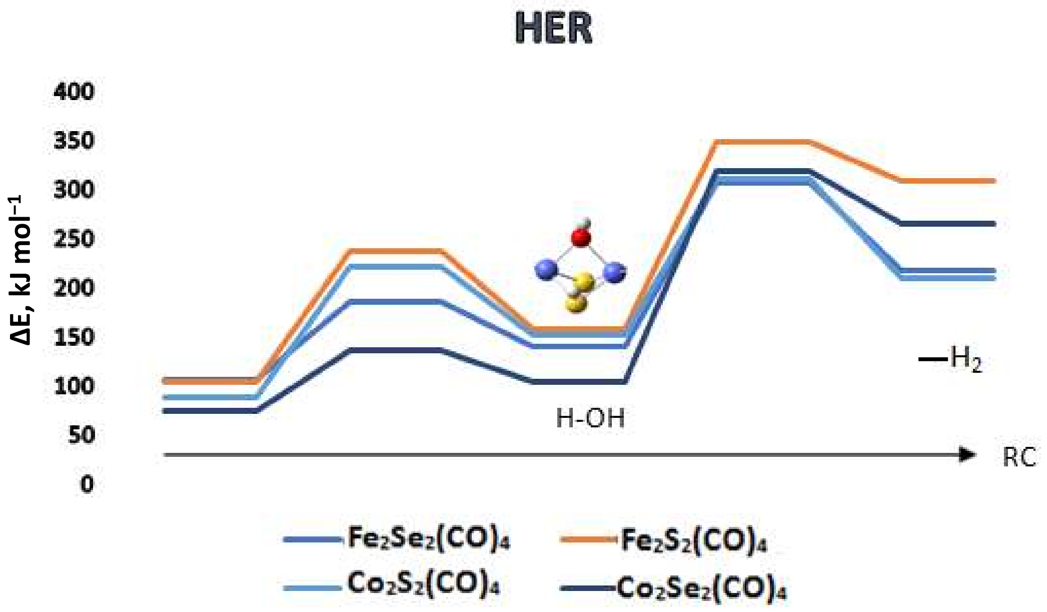

Figure S1. (a) The reaction path of water dissociation and hydrogen evolution (HER) on different tetracarbonyl complexes of iron and cobalt in their triplet states. ΔE is the energy difference relative to the global minima (singlet state) complexes; the global minima are reference zero. RC – reaction coordinate.

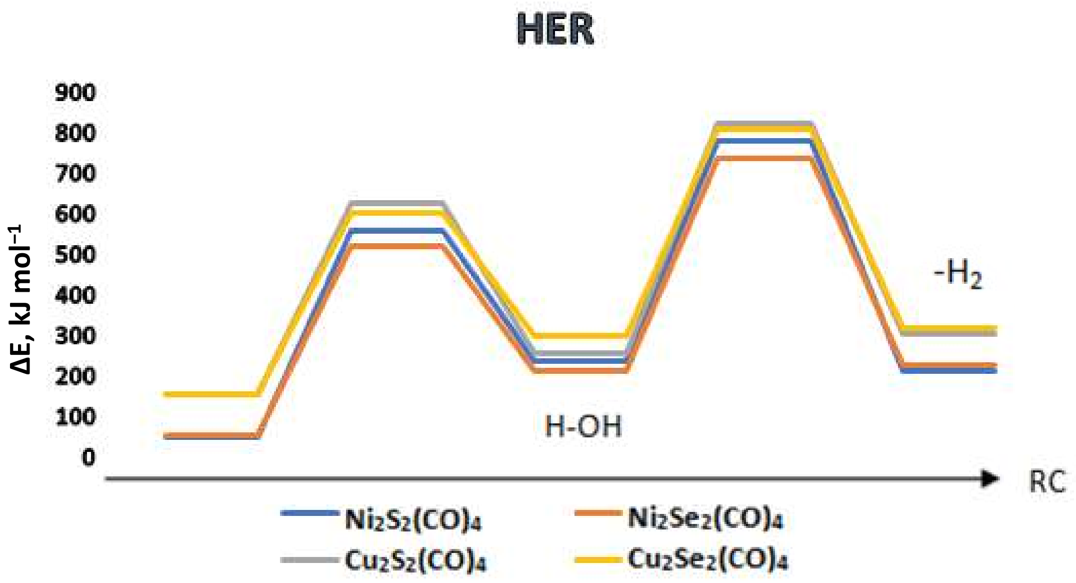

Figure S2. The reaction path of water dissociation and hydrogen evolution (HER) on different tetracarbonyl complexes of nickel and copper in their triplet ground states. ΔE is the energy difference relative to the ground state of the complexes; the global minima are reference zero. RC – reaction coordinate.

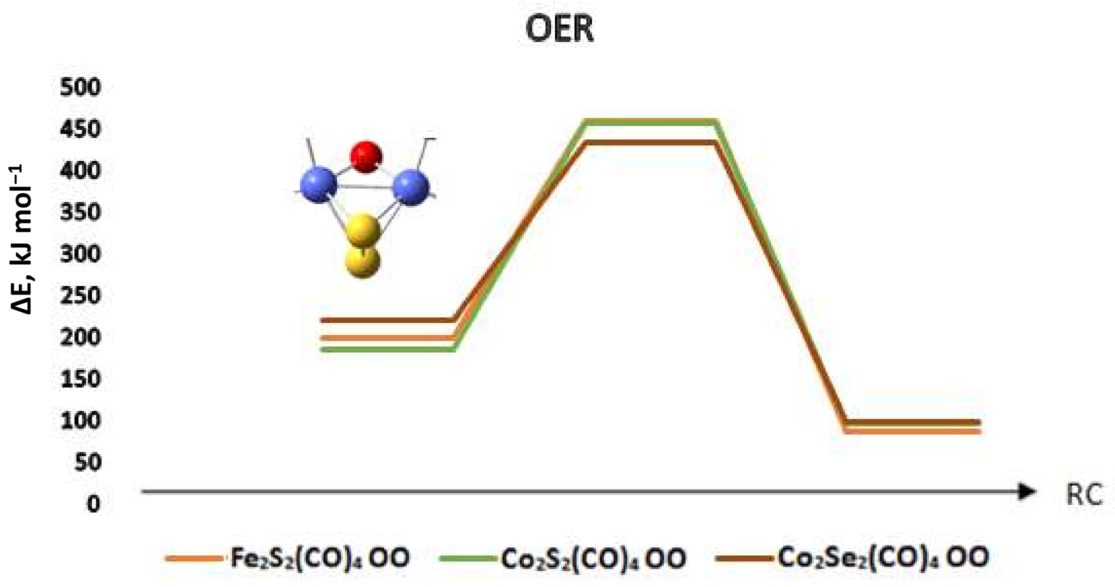

Figure S3. The reaction path of oxygen formation and oxygen evolution for cobalt and iron chalcogenide complexes in their triplet states. ΔE is the energy difference relative to the ground state complexes; the global minima are reference zero. RC – reaction coordinate. This pathway is followed with spin-binding between dioxygen and the triplet state of the complexes.

Table S1. Energy gaps between singlet and triplet states minima

Complex	Singlet to triplet excitation energies, kJ mol^{-1}
$Co_2S_2(CO)_4$ afm singlet	87
$Co_2Se_2(CO)_4$ singlet	74
$Fe_2S_2(CO)_4$ afm singlet	104
$Fe_2Se_2(CO)_4$ singlet	106
$Ni_2S_2(CO)_4$ afm singlet	47
$Ni_2Se_2(CO)_4$ singlet	53
$Cu_2S_2(CO)_4$ singlet	156
$Cu_2Se_2(CO)_4$ singlet	152

Afm – antiferromagnetic state

Cartesian Coordinates of ground state $Co_2S_2(CO)_4$, rhombic core (Figure la in main text)

Co,0,-1.4540460477,0.2802719304,-0.2079896802

S,0,-0.0000472712,1.9034076554,-0.6875643344
S,0,-0.0000183429,0.2299415213,-1.8989343576
Co,0,1.4539905803,0.2803095856,-0.2079761468
C,0,-2.3362693124,0.8867213576,1.1943803678
C,0,-2.3339883212,-1.2424764984,-0.3454388787
C,0,2.336186676,0.8867720598,1.1944054706
C,0,2.3339834335,-1.2424097843,-0.3454157758
O,0,-2.8991074208,1.2891028709,2.0999858646
O,0,-2.8951356883,-2.2295066568,-0.4442851063
O,0,2.8990101556,1.2891565048,2.1000187181
O,0,2.8951685593,-2.2294195463,-0.4442521414

Cartesian Coordinates of ground state $Co_2S_2(CO)_4$, planar core, Figure 1b in main text
Co,-1.2711761895,-0.0507291076,-0.0958446513
C,-2.4286971523,-0.9925710949,-1.0635105341
C,-2.4648952181,0.8342716056,0.8815262889
O,-3.1597789308,-1.5307702383,-1.7448767271
S,-0.0448839775,1.7185386329,-0.0384189569
S,0.0390141358,-1.7584759964,-0.1635971065
O,-3.2155150536,1.3366003289,1.569010691
Co,1.2654063509,0.0107135505,-0.1056697774
C,2.4141498573,1.0205658644,-1.0134280955
C,2.4681528731,-0.942096773,0.7937515942
O,3.1390451496,1.6070812776,-1.6606727527
O,3.2252591952,-1.4928389196,1.4355132373

Cartesian Coordinates of ground state $Fe_2S_2(CO)_4$, rhombic core
Fe,0.1943278,1.5512730841,0.
S,1.5066405493,-0.0128583199,-1.0447789887
S,1.5066405493,-0.0128583199,1.0447789887
Fe,0.1863233266,-1.5702396028,0.
C,-0.5570114885,2.5042301832,-1.3233309611
C,-0.5570114885,2.5042301832,1.3233309611
C,-0.5707414152,-2.5189442771,1.3230937834
C,-0.5707414152,-2.5189442771,-1.3230937834
O,-1.0329989368,3.1113858543,-2.1659659683
O,-1.0329989368,3.1113858543,2.1659659683
O,-1.0506597269,-3.1234615263,2.165398544
O,-1.0506597269,-3.1234615263,-2.165398544

Cartesian Coordinates of ground state $Fe_2S_2(CO)_4$, planar core
Fe,-0.1687659488,1.8507939129,0

C,-0.0347570359,3.1323622678,1.3894285023
C,-0.0347570359,3.1323622678,-1.3894285023
O,0.042274759, 3.8680203935,2.2476302562
S,-0.3372969584,0.2347537736,-1.5263179723
S,-0.3372969584,0.2347537736,1.526317972
O,0.042274759, 3.8680203935, -2.2476302562
Fe,-0.5056679284,-1.3813167697,0.
C,-0.6393268009,-2.6631268746,1.3892861671
C,-0.6393268009,-2.6631268746,-1.3892861671
O,-0.7160800266,-3.3989941319,2.2473303529
O,-0.7160800266,-3.3989941319,-2.2473303529

Cartesian Coordinates of ground state $Co_2Se_2(CO)_4$ rhombic core
Co,-0.2138119916,0.2777842134,-0.2381251148
Co,-0.1983067797,0.2890178196,2.7578074131
Se,1.5668170522,-0.1632490132,1.2524300432
Se,-0.3270112047,-1.5408694569,1.2672181871
C,-1.736576051,0.1343111799,-1.1072781555
C,0.382075566,1.6757706734,-1.1243048742
C,0.4070980806, 1.6936006154, 3.626753761
C,-1.7118433066,0.1525341603,3.6440036574
O,-2.7241337673,0.0351713727,-1.6689558348
O,0.7743675927,2.580507492,-1.6971268984
O,0.8055013042,2.6026229828,4.1886996074
O,-2.693396135,0.0578901571,4.2168589206

Cartesian Coordinates of ground state $Co_2Se_2(CO)_4$ planar core
Co,0,-0.2491570819,0.3250985776,-0.3325074715
Co,0,-0.230854853,0.334358997,2.7905213705
Se,0,1.5152123535,0.2804380218,1.233868777
Se,0,-0.7334320851,-1.3552805032,1.2518965884
C,0,-1.819103088,-0.0085848099,-1.2641302192
C,0,0.5418642866, 1.70883519, -1.2830623895
C,0,0.4394967167, 1.6417016942, 3.7641132514
C,0,-1.6707959293,0.1066258337,3.781027323
O,0,-2.7876240185,-0.2397069345,-1.8016812178
O,0,1.0542853584,2.5549830444,-1.8324891253
O,0,0.8759969513,2.4894296002,4.3913715916
O,0,-2.6051082511,-0.0428065147,4.419273848

Cartesian Coordinates of Configurations displayed on Figure 3b-f

$Co_2S_2(CO)_4$ [H^+,e^-] at S

Co,0,-1.4137563834,0.2963422428,-0.1741681571
S,0,-0.0261071079,1.9968393363,-0.5456733938
S,0,-0.0490499913,0.5291122942,-1.9819740507
Co,0,1.2228211682,0.2334331382,-0.0460220543
C,0,-2.4670726683,0.9198358408,1.1568820814
C,0,-2.1387314968,-1.3272592908,-0.3570944587
H,0,-0.2121511446,-0.3788233721,0.6902986818
C,0,2.3552132671,0.8737929236,1.158194187
C,0,1.9994383145,-1.3528445618,-0.2079620903
O,0,-3.0828040581,1.2948777489,2.0351041674
O,0,-2.5666614083,-2.3742364096,-0.4656593414
O,0, 3.0660766823, 1.2863661381,1.950116065
O,0,2.4849337367,-2.3764797086,-0.3292561262

$Co_2S_2(CO)_4$ [H^+,e^-] at Co

Co,0,1.3841230347,0.1878463517,-0.0717656184
C,0,2.8795757042,1.1960592662,-0.3504919009
C,0,2.0981644944,-1.1482201043,0.9929536747
O,0,3.7875128216,1.8352263052,-0.5737389036
S,0,0.0717063132,1.7802721888,-0.5905014527
S,0,0.2589634428,-1.2468403637,-1.4607867956
H,0,0.1975951948,-0.4204673489,-2.5220104892
O,0,2.5091850381,-1.9502925643,1.6791768953
Co,0,-1.2938249207,0.0591650584,-0.2956776143
C,0,-2.4310506048,1.1372833813,0.5192049983
C,0,-2.2664048429,-1.3947705367,0.0179782502
O,0,-3.1644274463,1.8364808843,1.038655715
O,0,-2.882759199,-2.3288298681,0.2342624011

$Co_2Se_2(CO)_4$ [H^+,e^-] at S

Co,1.3350786045,0.0000563955,0.2108012916
Se,-0.0000176296,1.1612147852,-1.3888504818
Se,0.0000234312,-1.160770961,-1.389419187
Co,-1.3350830286,0.0000371643,0.210791381
C,2.2308172625,-1.327956361,0.9726221617
C,2.2315432304,1.3278883423,0.9721661155
H,-0.0000082496,-0.0000590081,1.207089708
C,-2.2314354514,1.3278408094,0.9723223027
C,-2.2309337835,-1.3280079212,0.9724438627
O,2.780761024,-2.1887398949,1.477400266
O,2.7817622583,2.1885465398,1.4768644082
O,-2.7816279589,2.1884688214,1.4770992692

O,-2.7809127093,-2.1888152917,1.4771439523

$Co_2Se_2(CO)_4$ [H^+,e^-] at Co_2

Co,-1.4735294889,0.2564282059,0.2615476982
Se,-0.1230871324,-0.0379971645,-1.6919792013
Se,0.2155403649,1.893788475,-0.3982711097
Co,1.426870445,-0.0701784927,0.0903186081
C,-3.1243249578,1.0368466087,-0.0950752115
C,-1.5196383607,0.0411368347,2.0702401622
H,-2.1000586379,-1.0780316518,0.1726157434
C,1.9875288189,-1.7391489848,0.0505895753
C,2.4139049916,0.4470227607,1.452618703
O,-4.1599102085,1.4355541366,-0.3291975883
O,-1.5701498903,-0.1509742533,3.188453284

O,2.3420482418,-2.8222582515,0.0225789597
O,3.0520080044,0.788215017,2.334011487

$Co_2Se_2(CO)_4$ [H^+,e^-] at Co

Co,0,-0.1440990554,0.2430142967,1.4085408178
Co,0,-0.0995099866,0.1599461686,-1.417491595
Se,0,-1.9117579965,-0.2260166831,0.0235437812
Se,0,1.1226808351,-1.2740671148,0.0720269462
C,0,1.2945968771,1.1532346235,2.1066409161
C,0,-1.3927516496,0.4797001715,2.7110583749
C,0,-1.1838186893,1.008652054,-2.511213074
C,0,1.3468835823,0.5633610264,-2.3440112347
O,0,2.1748142198,1.749378739,2.4987550639
O,0,-2.1662460767,0.5992043986,3.5308228981
O,0,-1.8849960708,1.5494523977,-3.229753713
O,0,2.2831272591,0.8451064655,-2.9341933873
H,0,2.4979517501,-0.7228185661,0.0852742061

$Co_2S_2(CO)_4$ [H^+,e^-] at Co, midway

Co,-0.0327577061,0.1166133898,1.418457858
S,1.2248448926,1.4027995656,0.0486652096
S,-1.65320562,0.5314893476,0.0439846783
Co,0.0001298214,0.1284653626,-1.3869187204
H,2.4730418461,0.8832646017,0.0569233878
C,1.3617045128,-0.8207742728,2.1864541741
C,-1.349935932,-0.1758850028,2.6393390993

C,1.3989011672,-0.2730031468,-2.3913800067
C,-1.1388225244,-0.6951500363,-2.4483860532
O,2.2123652167,-1.4441858504,2.5984637127
O,-2.1794256719,-0.3262528799,3.3953997932
O,2.3075358574,-0.5455776971,-3.0266616366
O,-1.8821460258,-1.2198813783,-3.1343414961

Figure 4,5 Cartesian Coordinates of complexes with lowest energy pathways

Water adsorbed
Fe,0,-0.6283073865,1.2519805185,0.1799140703
C,0,-0.0928939963,2.4039188304,1.4688534082
C,0,-0.0071443666,2.2272336436,-1.1554256955
O,0,0.2590579842,3.1423581156,2.2589041527
S,0,-1.4278569324,-0.1653998427,-1.5383402562
S,0,0.8258842018,-0.1548886988,0.8661324854
O,0,0.4671113389,2.8257640396,-2.0009049494
Fe,0,-0.5283858933,-1.4865739407,-0.131941859
C,0,-0.5878060731,-2.6272645604,1.2684256959
C,0,0.3613508628,-2.5910794226,-1.1710279953
O,0,-0.6842677218,-3.3550739986,2.1403984725
O,0,0.9879477844,-3.2625997929,-1.8472102421
O,0,-2.668851747,1.8260177542,0.3126772194
H,0,-2.9400551534,2.7192585957,0.0699280231
H,0,-3.0403429817,1.2166877989,-0.35175244

TS1

$Co_2S_2(CO)_4 \cdot H_2O$

C,-0.0657153164,2.9785195414,1.2353261318
C,-0.5839855971,2.6450893677,-1.2786382004
O,-0.0984776071,3.8388865202,1.9796238151
S,0.9896645993,0.2834613703,-1.4630757513
S,0.2894520119,0.2222092751,1.621989403
O,-1.0891398544,3.2375494595,-2.1073881705
Co,-0.0088105129,-1.2126617882,0.0518563877
C,-0.747536139,-2.3015171852,1.3132991334
C,-0.6864702681,-2.0893565344,-1.4670484028
O,-1.1598287667,-2.99176923,2.1090826442
O,-1.0865432218,-2.5678493979,-2.4083630926
O,1.8104000211,-1.9237650466,-0.0735176449
H,1.8835064279,-0.7133353046,-0.7468910706
H,2.2335944469,-1.816692721,0.7876065612

$Co_2Se_2(CO)_4 \cdot H_2O$

Co,0,-0.0056693739,0.3651256022,1.1816661115
Co,0,-0.0056693739,0.3651256022,-1.1816661115
C,0,-0.7536661747,0.0991146675,2.7960564081
C,0,-0.7536661747,0.0991146675,-2.7960564081
C,0,1.7158993582,0.5964419285,-1.6733688373
Se,0,0.7612468444,-1.5288623382,0.
Se,0,-2.0682984816,0.1136174897,0.
C,0,1.7158993582,0.5964419285,1.6733688373
O,0,-1.2136036057,-0.1481856171,3.8047303267
O,0,-1.2136036057,-0.1481856171,-3.8047303267
O,0,2.7937623963,0.7539778419,-1.9924167883
O,0,2.7937623963,0.7539778419,1.9924167883
O,0,-0.3087793445,2.1241400199,0.
H,0,-1.5764767119,1.7196250247,0.
H,0,0.0637248883,3.0186479215,0.

$Fe_2S_2(CO)_4{\cdot}H_2O$

Fe,0,1.1876285658,0.1063565748,-0.5083951088
C,0,2.5068135525,1.3253101792,-0.258554254l
C,0,2.0573169249,-1.1398117315,0.4041177819
O,0,3.3482152013,2.0702009164,-0.1068569007
S,0,-0.1363977079,1.3651376148,0.709084837
O,0,2.5877234204,-1.934131899,1.0196216671
Fe,0,-1.4534607356,-0.0123666382,-0.1814002668
C,0,-2.8317637106,1.0972617774,-0.3626008482
C,0,-2.3987497493,-1.1343093492,0.8218519972
O,0,-3.7181589716,1.7933421704,-0.5315110609
O,0,-2.9702426304,-1.809698536,1.5383077194
O,0,1.7261897426,-0.2802273214,-2.3600116144
H,0,1.9782503574,0.4922932617,-2.8795880033
S,0,-0.3659680761,-1.5924552198,-1.2680408957
H,0,0.4295438766,-0.9476925493,-2.3288982798

$Fe_2Se_2(CO)_4{\cdot}H_2O$

Fe,0,1.1839754241,0.1605782088,0.6693123205
Se,0,-0.5866486679,1.2177004645,-0.5513245163
Se,0,0.0267536025,-1.9963670326,0.0647778573
Fe,0,-1.735178721,-0.4144252677,0.5571371197
O,0,0.7805270465,1.1540401228,2.2027899101
C,0,2.2835550906,1.2790230864,-0.1417799982
C,0,2.4940279864,-0.7748596573,1.5040018329
H,0,0.2011742018,-2.7322917437,1.337155663

C,0,-2.8075519912,0.9083710483,1.1632114974
C,0,-2.9221526869,-1.0412079558,-0.5876562077
H,0,1.4827390869,1.218304068,2.8583255389
O,0,2.9975408453,1.9982356561,-0.6636095105
O,0,3.3327302846,-1.3418898046,2.0239073958
O,0,-3.4925784636,1.7285077114,1.5487338562
O,0,-3.6485054382,-1.3963811847,-1.3898203392

$Ni_2Se_2(CO)_4 \cdot H_2O$

Ni,0,1.2449466167,-0.3058265493,0.0578731079
Se,0,-0.2988297221,-1.499382221,1.3081277443
C,0,1.8278287209,-1.8860700977,-0.6522329118
Ni,0,-1.4260956435,-0.0603622433,-0.1080600565
H,0,-0.0331005465,-0.3271035023,2.5928264767
Se,0,1.833259275,1.1506572653,1.9344342878
C,0,1.9588218598,0.9139046442,-1.0595853531
O,0,2.1914228379,-2.8537311641,-1.1176000926
C,0,-1.0861069595,0.9913603663,-1.5156919289
C,0,-3.182105355,-0.0607236132,0.2799679643
O,0,0.4868034303,0.5949782727,3.1928741006
O,0,2.426345809,1.6778609139,-1.7556369698
O,0,-1.0257859057,1.6832309502,-2.4209681028
O,0,-4.2972763394,-0.044748043,0.5025851501
H,0,-0.1545194278,1.3176137614,3.260408023

$Cu_2S_2(CO)_4 \cdot H_2O$

Cu,0,-0.1330230928,0.0072856271,1.6805777494
C,0,0.8551102257,-0.1742848395,3.3567809288
C,0,-2.0396994243,0.2417133171,1.8587280893
O,0,1.5179436777,-0.358520057,4.2503297207
O,0,-3.1683718855,0.2635122371,1.8854645897
Cu,0,0.092554167,0.0849343291,-1.3987247527
C,0,1.1383489707,-0.0911873263,-3.0248590189
C,0,-1.8051289892,0.3260205563,-1.7107676989
O,0,1.8292251622,-0.2932341421,-3.8954452765
O,0,-2.93017276,0.3496847987,-1.8053989521
O,0,2.7136331752,-0.0952000219,0.5454182648
H,0,3.3236328554,0.0065496059,-0.1997648828
S,0,1.1046804827,1.3896579619,0.2338548403
S,0,0.2709798508,-1.6857409911,0.1180207667
H,0,2.1096218544,-0.9008703753,0.3606964624

Reaction intermediate, water dissociation

$Fe_2S_2(CO)_4$, $H_2O \rightarrow$ H-OH

Fe,0,-1.0686242806,-1.264922385,2.3920151626
C,0,-1.422135816,-2.19682536,3.9089997395
C,0,0.0969356956,-2.4582787651,1.6674163626
O,0,-1.6557188698,-2.7837363719,4.8538325385
O,0,0.7991249876,-3.217175481,1.1943810373
Fe,0,-1.0695821147,1.2641089394,2.391940453
C,0,-1.4237940236,2.1958317503,3.9088725185
C,0,0.0950724727,2.4583041806,1.6672678226
O,0,-1.6578089622,2.7826334001,4.8536663118
O,0,0.7966794071,3.217713061,1.1941903417
O,0,0.2556525393,0.0001135793,3.0223759236
H,0,0.3497019307,0.0001769389,3.9810925664
S,0,-2.8612100166,-0.0010685333,2.9707006437
S,0,-1.1751111284,-0.0005039841,0.4574210116
H,0,0.0886628089,-0.0000391191,-0.0305359036

$Fe_2Se_2(CO)_4$, H_2O → H-OH

Se,0,0.0001055464,1.3678922938,-1.1440656168
Se,0,0.0001509433,-1.7116833429,-1.2601067652
Fe,0,1.5449890687,-0.1628279741,-0.1279793526
Fe,0,-1.54491389,-0.1628752724,-0.1282199848
H,0,0.0001012433,-2.8892859599,-0.3736919064
O,0,0.0001100704,2.9360061679,-0.1826530219
C,0,2.5746156672,-1.4906238738,0.5150270071
C,0,2.5096184723,1.1277874272,0.6673386915
C,0,-2.5747251359,-1.4907031358,0.5144319936
C,0,-2.5095849431,1.1277096562,0.6670936263
H,0,-0.0001487778,3.6528479303,-0.8293346104
O,0,3.2333908566,-2.330108259,0.9238928272
O,0,3.1309338316,1.9375851087,1.1773798713
O,0,-3.2337112162,-2.330119069,0.9230970626
O,0,-3.1309318113,1.9374736845,1.1771508036

$Co_2Se_2(CO)_4$, H_2O → H-OH

Co,0,-0.1586024894,0.5533192644,0.1642760322
Co,0,-0.1586024894,0.5533192644,2.4910339678
Se,0,1.7379460995,1.489473053,1.327655
Se,0,0.3148277582,-1.4870477138,1.327655
C,0,-1.6443807976,-0.316069685,-0.341690112
C,0,0.4778145756,0.8911158981,-1.4812514437

C,0,0.4778145756,0.8911158981,4.1365614437
C,0,-1.6443807976,-0.316069685,2.997000112
O,0,-2.5941886953,-0.8376231321,-0.6817532368
O,0,0.9182533492,1.0639182332,-2.5142937853
O,0,0.9182533492,1.0639182332,5.1696037853
O,0,-2.5941886953,-0.8376231321,3.3370632368
H,0,2.6559437761,0.3355208107,1.327655
O,0,-0.9259850634,2.0035209259,1.327655
H,0,-1.8814904838,2.1202238267,1.327655

$Co_2S_2(CO)_4$, H_2O → H-OH

Co,0,-1.0430200233,-1.3688779787,2.4506833385
C,0,-1.4740833494,-2.5025295651,3.8036344133
C,0,0.1092183145,-2.5065502209,1.6040966678
O,0,-1.7626448314,-3.1817952099,4.6662260071
O,0,0.8393120074,-3.1865827451,1.0644614258
Co,0,-1.0440589721,1.368090187,2.4506107017
C,0,-1.4759875573,2.5014963977,3.8034840529
C,0,0.1073093857,2.5065913922,1.6039685323
O,0,-1.7650697501,3.1806014159,4.6660286034
O,0,0.8368833337,3.187153912,1.0642971106
O,0,0.3318224834,0.0001267989,3.1249220475
H,0,0.2482861392,0.0001265338,4.0846638107
S,0,-2.564486812,-0.0009529438,3.2698174913
S,0,-1.2191512761,-0.0005102284,0.6582879758
H,0,0.0235155378,-0.0000558996,0.1184543513

HER, TS2

$Co_2S_2(CO)_4$, H_2O → H_2

Co,0,0.1104707063,1.6610114136,0.0517464568
C,0,-0.0657153164,2.9785195414,1.2353261318
C,0,-0.5839855971,2.6450893677,-1.2786382004
O,0,-0.0984776071,3.8388865202,1.9796238151
S,0,0.9896645993,0.2834613703,-1.4630757513
S,0,0.2894520119,0.2222092751,1.6219894031
O,0,-1.0891398544,3.2375494595,-2.1073881705
Co,0,-0.0088105129,-1.2126617882,0.0518563877
C,0,-0.747536139,-2.3015171852,1.3132991334
C,0,-0.6864702681,-2.0893565344,-1.4670484028
O,0,-1.1598287667,-2.99176923,2.1090826442

O,0,-1.0865432218,-2.5678493979,-2.4083630926
O,0,1.8104000211,-1.9237650466,-0.0735176449
H,0,1.8835064279,-0.7133353046,-0.7468910706
H,0,2.2335944469,-1.816692721,0.7876065612

$Co_2Se_2(CO)_4$, $H_2O \rightarrow H_2$

Co,0,0.0125166292,0.1380436785,1.2232412364
Co,0,-0.0027847887,0.1724388111,-1.1093428843
Se,0,-1.7900310416,-0.8187127816,0.0497174413
Se,0,0.2479652124,-1.9777346722,0.0063727074
C,0,1.719171044,0.4631438394,1.7217263858
C,0,-0.7201694265,0.2677848161,2.8632108256
C,0,-0.6357063873,0.3157401483,-2.7850648043
C,0,1.7503457803,0.6907082735,-1.5593349066
O,0,2.7819752939,0.6196920698,2.0813308372
O,0,-1.2039417504,0.3678681367,3.8857369344
O,0,-1.0653958538,0.4278857141,-3.8301764694
O,0,2.7608210409,0.5848459107,-2.0818604973
H,0,1.7343670677,2.3205118266,-0.5748138677
O,0,-0.2442918555,1.6152700661,0.038239682
H,0,0.9014029356,2.2052300629,-0.2237759306

$Fe_2Se_2(CO)_4$, $H_2O \rightarrow H_2$

Fe,0,1.2734015768,0.0963487767,0.100025288
C,0,1.887080599,1.7426749699,-0.4170592479
C,0,2.5735394906,-0.8739175806,-0.7149304367
O,0,2.2338433391,2.7637502807,-0.7708174999
S,0,-0.0027158199,0.5185924369,1.9382603381
O,0,3.3939963632,-1.4964665556,-1.1919575582
Fe,0,-1.2623214675,0.0868265867,0.0909588428
C,0,-1.8847215911,1.7284060157,-0.4306743768
C,0,-2.5493728141,-0.8930957957,-0.7330973892
O,0,-2.2366598752,2.7468560946,-0.786890159
O,0,-3.3618396517,-1.5218558419,-1.2156391313
O,0,0.0112663426,-0.1117829538,-1.2847484321
H,0,0.0158382287,-1.3730745044,-1.3084711517
S,0,0.0083730808,-1.5158948368,0.9977628246
H,0,0.0180539887,-2.2821844825,-0.9138441907

$Fe_2Se_2(CO)_4$, $H_2O \rightarrow H_2$

H,0,-0.8839623232,-2.2615840776,-1.1074633255
O,0,-0.0553679795,-0.2576545594,-1.0202589697

H,0,-0.3598355307,-1.4551862101,-1.1971637505
Fe,0,1.3063399215,0.103925968,0.2120963287
Fe,0,-1.3633241856,0.0793703902,0.2487089013
Se,0,-0.0461745264,1.6545958659,1.2937381259
C,0,2.211 9567644, 1.0827526726, -1.0220271026
C,0,2.2021 545244, -1.4517348843, -0.02774043
C,0,-2.4131768751,0.951537496,-0.9338085459
C,0,-2.118179767,-1.6381057178,-0.0178235022
O,0,2.756817222, 1.6998328496, -1.8024616796
O,0,2.7819667704, -2.4193149686,-0.158179342
O,0,-3.0601444763,1.5087472028,-1.6840838158
O,0,-2.9020018421,-2.4573877629,0.1821741666

OER

$Co_2Se_2(CO)_4$ – OOH*

Co,0,0.2606287833,0.4141048236,-1.5226563364
Co,0,0.0564205547,-0.0895535484,1.4742633442
C,0,-0.7988898334,0.0654601416,-3.0231284424
C,0,-0.8099514363,0.8778742355,2.7562417271
C,0,1.7835319791,-0.1418511104,2.1103645813
Se,0,0.2418368695,-1.7724201409,-0.2581668296
Se,0,-1.8653035556,-1.0392675333,0.4649416618
C,0,1.9677470399,0.6114057987,-2.3262807901
O,0,-1.4307278706,-0.1451472021,-3.9438102369
O,0,-1.3528459499,1.4972351897,3.5341772549
O,0,2.8372590411,-0.2048028216,2.5176989965
O,0,2.9923634476,0.682113897,-2.8069849758
O,0,1.5417517231,2.2297573148,0.3415483348
H,0,1.1979776511,3.1202554208,0.4959880547
O,0,0.2954224264,1.491645935,0.1272337559

$Fe_2Se_2(CO)_4$ – OOH*

Fe,0,1.4527268857,-0.0790597928,0.212200546
O,0,0.2254738449,-0.5212219987,1.5915585736
Se,0,0.0216527192,1.3460467065,-1.1261349093
Fe,0,-1.4243622298,-0.0790356251,0.3092195769
O,0,0.4297797825,0.1840388383,2.8352429092
Se,0,-0.0301959436,-0.9823846404,-1.4942578544
C,0,2.4518955574,1.1557915863,1.1062472854
C,0,2.5219331395,-1.5369011441,0.5277489878
C,0,-2.4454111571,1.1119367059,1.1545572867
C,0,-2.534284574,-1.4385366124,0.647522292
H,0,-0.2804998712,0.8426740146,2.8220820159
O,0,3.1011478811,1.9303106957,1.6229322279
O,0,3.1760874604,-2.4429149632,0.7191319142

O,0,-3.1236454745,1.8809188711,1.6666760681
O,0,-3.2540754105,-2.3034905419,0.8475763001

$Fe_2Se_2(CO)_4$ – OO*

Fe,0,1.4598475396,-0.0392833625,0.3792655153
O,0,0.3914502257,-0.2125935611,2.0401375196
Se,0,-0.0274676274,1.2800885635,-1.0377604743
Fe,0,-1.7554037925,-0.0102201394,0.0771337772
O,0,0.8247044078,-0.2468949567,3.1984036034
Se,0,-0.0288454594,-1.0741510509,-1.256529453
C,0,2.5804553353,1.2548071484,0.9448079879
C,0,2.5884811268,-1.4106784554,0.6881267037
C,0,-2.7949569691,1.2466038682,0.8191465854
C,0,-2.7934922244,-1.3827044043,0.5762137743
O,0,3.310414753,2.072953268,1.2539790979
O,0,3.3229975423,-2.2686143776,0.837346014
O,0,-3.4655534758,2.047408427,1.2828889916
O,0,-3.462647042,-2.2559184273,0.8857093079

$Fe_2Se_2(CO)_4$ – OO*

Co,0,-0.0094999369,0.0149718442,1.4652187336
C,0,-1.4198641649,-0.5878784547,2.4673651781
C,0,1.4066112077,-0.5603694398,2.4735270092
O,0,-2.3021261353,-0.9560743179,3.0720989248
Se,0,-1.4203998831,1.2600001601,-0.0092354022
O,0,2.2931283272,-0.911857853,3.0822868915
Co,0,-0.0069206728,-0.006247522,-1.4630245915
C,0,-1.4156192733,-0.6241929873,-2.45834370
C,0,1.410902462,-0.5962595576,-2.460384000
O,0,-2.2969778843,-1.0017400004,-3.0586048803
O,0,2.2984999109,-0.9566710384,-3.0623148382
O,0,0.0068391514,-1.4817923422,0.0117642801
O,0,-0.0843358425,-2.775693584,0.0208692983
Se,0,1.3848952238,1.278681813,-0.0069738746
